# Cell-Free DNA Plasma Levels Differ in Age-Specific Pattern in Healthy Rats and Castrates with Testosterone-Induced Benign Prostatic Hyperplasia

**DOI:** 10.1155/2019/8173630

**Published:** 2019-06-02

**Authors:** I. N. Vasilyeva, V. G. Bespalov, J. D. Von, A. L. Semenov, G. V. Tochilnikov, V. A. Romanov, I. K. Alvovsky, D. A. Baranenko

**Affiliations:** ^1^Scientific Laboratory of Cancer Chemoprevention and Oncopharmacology, N.N. Petrov National Medical Research Center of Oncology, Pesochny, St. Petersburg, Russia; ^2^International Research Centre “Biotechnologies of the Third Millennium”, ITMO University, St. Petersburg, Russia

## Abstract

The purpose of this work was to study changes in the level of cell-free DNA (cfDNA) in the blood of young and old rats in the normal state and with induced benign prostatic hyperplasia (BPH). Male Wistar rats were divided into 4 groups—young (3 months), old (20 months), intact, or with testosterone-induced BPH. Groups with BPH were subjected to surgical castration and administration of testosterone esters at a dose of 25 mg/kg for a total of 7 injections for 20 days. In intact animals, the level of cfDNA in old rats (2.00 ± 0.14 ng/*μ*l) was significantly higher than that in the young (1.02 ± 0.30 ng/*μ*l). The body and the prostate weights of old rats were 1.6 and 1.4 times larger than those of the young, without an increase in the prostate index (PI). The testosterone level in the blood of young rats was 1.6 times higher than that of old (6.20 ± 0.93 nmol/l vs. 3.77 ± 0.55 nmol/l; NS). In animals with BPH, the level of cfDNA in old rats (3.14 ± 0.76 ng/*μ*l) was significantly higher than that in young rats (0.80 ± 0.14 ng/*μ*l). The body and the prostate weights in old rats were 1.8 and 2.3 times larger, than those in young rats, with an increase in the PI. The level of testosterone in the blood of young (15.76 ± 0.51 nmol/l) and old (16.99 ± 1.1 nmol/l) rats was not significantly different. Morphological signs of BPH were observed in the prostate of both young and old rats. During the induction of BPH in the experiment, according to the level of cfDNA, cell death processes have not changed significantly in young rats but significantly increased in old rats. A similar trend was observed in the group of intact animals. The obtained data indicate that apoptosis processes are enhanced during the development of BPH despite the growth of tissues in the prostate itself.

## 1. Introduction

Benign prostatic hyperplasia (BPH) is a disease with a high prevalence and continuous increase—more than half of men over 50 experience symptoms of BPH, and by the age of 80, this figure rises to 80% [1]. The existing early diagnostics is not specific and, often, is subjective, which creates a need for additional instrumental examinations, imposing a significant burden on both the patient and the health care system [2]. As a noninvasive biomarker of BPH and prostate cancer, it is proposed today to determine the level of cell-free DNA (cfDNA) in peripheral blood [3, 4]. It is also known that cfDNA is successfully detected in 50% of plasma samples and more than 70% of urine samples and the specificity and sensitivity of such tests range from 80% to 99.9% [3, 5]. In comparison, the specificity of tests for the determination of prostate-specific antigen (PSA) does not exceed 70% [4].

CfDNA is a cell death product associated with apoptosis, necrosis, and phagocytosis. Prolonged circulation of cfDNA in the blood may indicate the presence of benign or malignant pathological foci in the body; the differentiation of which is associated with the measurement of the overall level and integrity of cfDNA [4, 6, 7]. In prostate cancer in particular and malignant neoplasms in general, there is a predominance of necrosis, which is associated with the release into the bloodstream of a large number of disordered cfDNA fragments of various lengths. At the same time, BPH is associated with processes of apoptosis, in which there is a predominance of cfDNA fragments in the range from 180 to 210 bp. It is known that the level of cfDNA in BPH is higher than that in the population of healthy men, but significantly lower than that among men with prostate cancer [4].

The purpose of this study was to investigate the changes in the level of cfDNA in the blood of young and old rats in normal conditions and with the induction of benign prostatic hyperplasia.

## 2. Materials and Methods

### 2.1. Study Animals

The study was performed on 45 male Wistar rats of two age groups: 3 and 24 months old and weighing 140-160 g and 300-350 g, respectively, purchased from the “Rappolovo” animal nursery (Russia, Leningrad Region). Animals were housed 5–7 in polypropylene cages under standard 24 h light-dark regimen (12L : 12D) at 22 ± 2°C with relative humidity at 50 ± 10%. Rats received standard laboratory crop from Laboratorkorm Ltd. (Moscow, Russia) and tap water ad libitum. All animal experiments were performed following the approval of the N.N. Petrov Research Institute of Oncology Ethic Committee within the frame of rules declared by the European Treaty ETS No. 123 and in accordance with the requirements of Directive 2010/63/EU.

### 2.2. Induction of Benign Prostatic Hyperplasia (BPH)

7 days after placement, animals in both age groups were randomized to the intact control group and the BPH induction group (4 groups in total). On the first day of the experiment, surgical castration was performed: orchidectomy was performed in sterile conditions under ether anesthesia, by an incision in the midline of the scrotum. After ligation of the spermatic cord and blood vessels, the testicles with appendages were removed. The stump of the spermatic cord was refueled through the inguinal canal into the abdominal cavity, after which the scrotum was sutured. Starting from the 7th day after orchidectomy and then every other day, animals received subcutaneous injections of testosterone (Omnadren 250, Jelfa, Poland) in a dose of 25 mg/kg body weight, a total of 7 injections for 20 days. Rats were sacrificed on the 7th day after the last injection (the 36th day after castration), the blood was taken to determine cfDNA and serum testosterone, and the prostate was extracted.

### 2.3. Analysis of the Prostate

A full autopsy was performed on all animals. The prostate was isolated as a complex of the dorsolateral prostate, ventral lobes, and seminal vesicles with anterior prostate lobes (coagulating glands). The prostates were cleared of other tissues, the lobes were divided and weighed, the dorsolateral section was weighed separately, ventral and anterior lobes pairwise, and then fixed in 10% buffered neutral formalin, and then standard histological processing was carried out. To evaluate the prostate enlargement, the prostatic index (PI) was calculated as the ratio of the dorsolateral and ventral prostate fraction weights in mg/100 g of body weight.

### 2.4. Histopathological and Morphological Analyses of Prostatic Tissue

Prostate tissue blocks were embedded in paraffin and cut at 5 *μ*m and then stained with hematoxylin-eosin.

### 2.5. Cell-Free DNA (cfDNA) Levels in Peripheral Blood

The content of cell-free DNA (cfDNA) in the blood plasma was determined by ELISA using the Cell Death Detection ELISA kit (Roche, Sigma-Aldrich). The definition is based on quantitative sandwich ELISA. Two mouse antibodies produced against DNA (single and double stranded) and histones (H1, H2A, H2B, H3, and H4), specifically binding mono- and oligonucleosomes derived from the nuclei of eukaryotic cells (cat. no. 11 774 425 001, https://sigma-aldrich.com). Absorption with a wavelength of 450 ± 10 nm was measured on a Microplate ChroMate Reader tablet (Awareness Technology Inc., USA). We measured the concentration of the DNA of the ELISA kit-positive control by spectrophotometer (NanoPhotometer N-50, Implen, Germany). By diluting this positive control, calibration curves were constructed to quantify the DNA in our samples.

### 2.6. Testosterone Levels in Peripheral Blood

The serum testosterone level was determined by ELISA using the DRG Testosterone ELISA Kit (DRG Instruments GmbH, Germany). Absorption with a wavelength of 450 ± 10 nm was measured on a Microplate Reader ChroMate tablet (Awareness Technology Inc., USA).

### 2.7. Statistical Analysis

Data are presented as mean ± SEM (standard error of the mean). The analysis was performed using the GraphPad Prism 7 software; the significance of the differences was estimated by Student's *t*-criterion. Values of *p* < 0.05 were considered statistically significant.

## 3. Results

**Figure 1:**
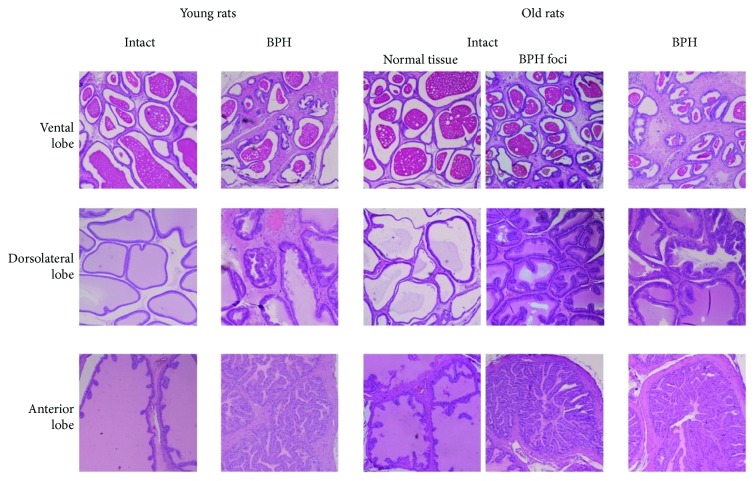
Histological features of BPH in old and young rats. *Young intact rats*: ventral prostate—smooth edges of acini, oval shaped with thin walls without folds, and evenly filled with secretion; dorsolateral prostate—smooth edges of acini, of arbitrary shape with thin walls, folding is allowed, and evenly filled with secretion; anterior prostate—large acini and folded two-layer walls. *Young BPH rats*: ventral prostate—a change in the cell walls of the acini, folding appears, and marked hyperplasia of muscle tissue; dorsolateral prostate—marked hyperplasia of the walls of the acini and muscle tissue; anterior prostate—the wall of the acini with marked hyperplasia. *Old intact rats*: ventral prostate—signs of BPH appear in the accumulation and ablation of acini and folding is detected; dorsolateral prostate—a low content of secretion and folding of the walls; anterior prostate—signs of BPH: focal high folding. *Old BPH rats*: ventral prostate—focal folding of acini and muscle hyperplasia; dorsolateral prostate—marked hyperplasia of muscle tissue and thickening of the walls; anterior prostate—the walls of the acini and muscle tissue with severe hyperplasia.

The results of the study are shown in Table 1. In the group of intact animals, the level of cfDNA in old rats was significantly higher than that in young rats (*p* < 0.05). The body weight of old rats was 1.6 times larger than that of the young (*p* < 0.001), and the prostate weight was 1.4 times larger. Differences in PI values in young and old rats were not statistically significant. The testosterone level in the blood of young rats was 1.6 times higher than that of the old (*p* < 0.05) (Table 1). A morphological examination of the prostate tissues of the young animals did not reveal the presence of BPH, while in old animals, focal hyperplasia was observed (Figure 1).

In the group of animals with benign prostatic hyperplasia, the level of cfDNA in old rats was similarly significantly higher than that in young rats (*p* < 0.001). The body weight of old rats was 1.8 times larger than that of the young (*p* < 0.001) and the prostate weight was 2.3 times larger (*p* < 0.001). The PI value of old BPH rats was statistically significantly higher than that of old intact rats (*p* < 0.05). The level of testosterone in the blood of young and old rats was not significantly different (Table 1). Morphological analysis of young and old animals confirmed the presence of BPH.

## 4. Discussion

We have found a significant increase in the level of cfDNA in old rats compared to young ones, which correlates with the literature data on age-related increases in the level of cfDNA in humans [8, 9]. It should be noted that age-related changes in rats are accompanied by a significant increase in the body weight. It has been established that the rate of apoptosis increases with age in many cell populations and organs, including the central nervous, cardiovascular, immune, and endocrine systems and the reproductive system [10]. It is believed that in older people, cfDNA is secreted not only due to systemic cell loss but also as a result of chronic inflammatory processes [9].

Today, it is known that the presence of chronic inflammation, both in humans and in animals, is a risk factor for precancerous and cancerous conditions in various organs, including the prostate. At the same time, the development of BPH is associated with an imbalance of cell proliferation and apoptosis [4]. It is believed that the basis for the development of BPH is a decrease in apoptosis in prostate tissues [11]. In particular, it has been shown that with age, atrophic acini in limited areas of the prostate epithelium persist without being subjected to apoptosis [11]. However, the presence in the tissues of a large number of defective cells contributes to the formation of a proinflammatory phenotype, causing the accumulation of immunocompetent cells in the tissues of the prostate, namely, T-lymphocytes and macrophages [12]. Epithelial and stromal cells of the prostate, as well as inflammatory cells, induce a local immune response. When the critical level of T-lymphocytes is reached, prostate cells are destroyed by CD8+ cytotoxic T-cells. This leads to the replacement of prostate tissue fibromuscular nodes and an increase in prostate size. Probably, in this case, fragments of the cfDNA of the substituted glandular cells circulate in the blood for a long time.

Another explanation for an increase in cell death and in the overall level of cfDNA is age-related circulatory disorders and local hypoxia, which lead to the formation of ischemic foci in the pelvic organs [13]. As a result, ischemic necrosis and concomitant apoptosis processes lead to the appearance of cfDNA [14] in the bloodstream.

**Figure 2:**
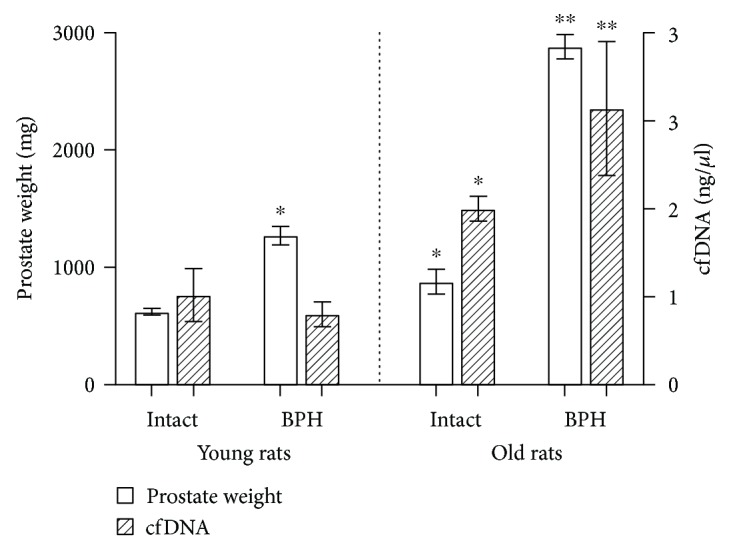
Prostate weight and cell-free DNA content. Statistical difference: ^∗^from intact young rats (BPH *p* < 0.001, intact old *p* < 0.05 for prostatic weight, and *p* < 0.05 for cfDNA); ^∗∗^from young BPH rats and intact old rats (prostatic weight *p* < 0.001 from intact old rats, *p* < 0.001 from young BPH, cfDNA *p* < 0.01 from young BPH, and *p* < 0.05 from intact old).

In our experiment, among old rats with the development of BPH, an increase in cfDNA level correlated with the largest increase in THE prostate weight (Figure 2) and prostatic index and was accompanied by histological changes in prostate tissue. It should be noted that already in intact old rats, signs of focal hyperplasia were observed and in old rats with BPH, hyperplasia progressed significantly. Thus, the accumulation of inflammation foci and remodeling of prostate tissues in a cohort of old rats led to the release of cfDNA into the systemic circulation; the increase in levels of which can be observed in our experiment.

On the other side, it is possible to speak about the hormonal nature of the development of BPH and its connection with THE natural aging processes. In our study, testosterone levels in old intact rats were statistically significantly lower than those in young intact animals; however, the level of cfDNA in young intact rats was significantly lower. Testosterone has been shown to protect cells from damage caused by oxidative stress [15]. At the same time, oxidative stress affects the accumulation of lipofuscin granules and can induce apoptosis in various cell types [15]. Thus, the natural decline in testosterone levels with age can cause an increase in apoptosis, which correlates with a high content of cfDNA in the blood.

Another important thing is that in young rats with BPH, the level of cfDNA was significantly lower than that in old rats, with a relatively equal level of testosterone. At the same time, the body weight and prostate weight in young rats were almost two times less than those in old rats. Thus, the effects of testosterone on old and young rats with the development of BPH differed significantly. Judging by the level of cfDNA, the processes of cell death did not change significantly after the action of testosterone in young animals and increased in old ones. Probably, in young rats, testosterone stimulates proliferative processes [16], thereby partially compensating for the concomitant BPH disorders [17].

We assume that an increase in the level of cfDNA in old rats is associated with activation of apoptosis accompanying the growth of prostate tissue, compared with young animals. On the one hand, the growth of prostate tissue during the development of BPH in old rats is possibly accompanied by the active secretion of small amounts of DNA [18], which then leads to a significant increase in cfDNA content by inducing apoptosis, as shown in vitro and in vivo [19]. On the other hand, an increased level of cfDNA in old rats, associated with increased apoptosis, as a result of intercellular signaling by soluble secretion factors, extracellular vesicles, and macrophages involved in apoptosis, leads to the formation of a niche for cell proliferation [20], causing enhanced prostate growth. It is possible that other cell death processes may be activated in old rats, for example, pyroptosis, which differs from apoptosis, necrosis, or oncosis, phagocytosis of cells and their remains, and also NETosis, which may not be accompanied by death of the neutrophils [18]. In continuation of our work, the study of DNA integrity patterns can be important for determining the source of cfDNA and for usage in diagnostics or treatment.

BPH and prostate cancer are a significant problem for an aging male population all over the world, and it is expected that the urgency of this problem will only increase due to the increase in life expectancy. However, the diagnosis of both benign and malignant prostate pathologies remains nonspecific and subjective, which requires additional instrumental studies to confirm the diagnosis. This not only affects the patient's quality of life and mortality but also carries an economic burden for the whole health care system. In particular, the specificity of the traditional PSA test can vary from 30% to 70%, in most cases, requires a biopsy, and is not able to differentiate between BPH and prostate cancer [21]. In addition, there is evidence of an increase in chronic inflammation, erectile dysfunction, and the development of lower urinary tract syndrome as a result of repeated biopsies [22]. In comparison, according to a number of researchers, the simplest PCR diagnostics of cfDNA, being a noninvasive technique, allows to achieve specificity and sensitivity of at least 70% and is capable of dividing benign and malignant pathologies [3, 4, 21].

## 5. Conclusion

The level of cfDNA in old and young rats differed significantly. Upon induction of BPH in the experiment, judging by the level of cfDNA, cell death processes did not change significantly in young animals but significantly increased in old animals. A similar trend was observed in the group of intact animals; however, it was significantly lower than that in BPH. Thus, in aging animals, cfDNA reflected the progression of BPH from partial atrophy of prostate tissues to progressive hyperplasia, which was accompanied by apoptosis and increased levels of cfDNA in the blood. Our data suggest that apoptosis is enhanced during the development of BPH despite the growth of tissues in the prostate itself. Considering that BPH is attributed to the diseases of aging men, the results confirm the need for further research of the diagnostic value of cfDNA as a noninvasive biomarker, which will provide patients with personalized diagnostics and more accurate treatment.

## Figures and Tables

**Table 1:** Summary analysis of indicators in the group of rats with BPH and in the intact control.

Indicator	Group and number of rats			
	Group 1 Young intact rats, *n* = 11	Group 2 Young BPH rats, *n* = 12	Group 3 Old intact rats, *n* = 10	Group 4 Old BPH rats, *n* = 12
Cell-free DNA (ELISA) (ng/*μ*l)	1.02 ± 0.30	0.80 ± 0.14	2.00 ± 0.14^vs1∗^	3.14 ± 0.76^vs2∗∗,vs3∗^
Body weight (g)	251.0 ± 5.5	237.0 ± 9.2	410.0 ± 9.8^vs1∗∗∗^	436.0 ± 15.7^vs2∗∗∗^
Prostate weight (mg)	623.0 ± 27.4	1273.0 ± 79.1^vs1∗∗∗^	879.0 ± 104.3^vs1∗^	2884.0 ± 102.9^vs2∗∗∗,vs3∗∗∗^
Prostatic index	248.0 ± 10.3	540.0 ± 36.5^vs1∗∗∗^	213.0 ± 23.1	669.0 ± 30.4^vs2∗,vs3∗∗∗^
Testosterone (nmol/l)	6.20 ± 0.93	15.76 ± 0.51^vs1∗∗∗^	3.77 ± 0.55^vs1∗^	16.99 ± 1.1^vs3∗∗∗^

The reliability of differences is marked: vs1: with group 1; vs2; with group 2; vs3: with group 3. ^∗^*p* < 0.05, ^∗∗^*p* < 0.01, and ^∗∗∗^*p* < 0.001.

## Data Availability

The data used to support the findings of this study are available from the corresponding author upon request.
